# Seagrass-derived carbon suppresses nitrification and N_2_O emissions by Comammox and canonical nitrifier communities

**DOI:** 10.1093/ismeco/ycag220

**Published:** 2026-07-29

**Authors:** Ruilin Su, Pui Ting Chan, Xiaodong Zhang, Jiawei Chen, Sangwook Lee, Xinlei Yang, Hoi Yau Chan, Yuxuan Lin, Jiying Li, Qingyun Yan, Hongbin Liu, Zhili He

**Affiliations:** Department of Ocean Science, Hong Kong University of Science and Technology, Hong Kong 999077, China; Marine Synthetic Ecology Research Center, Southern Marine Science and Engineering Guangdong Laboratory (Zhuhai), the China-ASEAN Belt and Road Joint Laboratory on Mariculture Technology, Sun Yat-sen University, Zhuhai 519082, China; Department of Ocean Science, Hong Kong University of Science and Technology, Hong Kong 999077, China; Department of Ocean Science, Hong Kong University of Science and Technology, Hong Kong 999077, China; Department of Ocean Science, Hong Kong University of Science and Technology, Hong Kong 999077, China; Department of Ocean Science, Hong Kong University of Science and Technology, Hong Kong 999077, China; Marine Synthetic Ecology Research Center, Southern Marine Science and Engineering Guangdong Laboratory (Zhuhai), the China-ASEAN Belt and Road Joint Laboratory on Mariculture Technology, Sun Yat-sen University, Zhuhai 519082, China; Department of Ocean Science, Hong Kong University of Science and Technology, Hong Kong 999077, China; Department of Ocean Science, Hong Kong University of Science and Technology, Hong Kong 999077, China; Department of Ocean Science, Hong Kong University of Science and Technology, Hong Kong 999077, China; Marine Synthetic Ecology Research Center, Southern Marine Science and Engineering Guangdong Laboratory (Zhuhai), the China-ASEAN Belt and Road Joint Laboratory on Mariculture Technology, Sun Yat-sen University, Zhuhai 519082, China; Department of Ocean Science, Hong Kong University of Science and Technology, Hong Kong 999077, China; Marine Synthetic Ecology Research Center, Southern Marine Science and Engineering Guangdong Laboratory (Zhuhai), the China-ASEAN Belt and Road Joint Laboratory on Mariculture Technology, Sun Yat-sen University, Zhuhai 519082, China

**Keywords:** Comammox, canonical nitrifiers, seagrass sediment, nitrous oxide, nitrification, sugar transport, metabolism

## Abstract

Seagrass meadows are critical regulators of coastal biogeochemical cycling, particularly through the exudation and accumulation of organic carbon that enriches their rhizosphere far beyond ambient marine levels. Microbially driven nitrification is a key process controlling nitrous oxide (N_2_O) emissions, yet the ecological roles of complete ammonia oxidizers (Comammox) and canonical nitrifiers in seagrass ecosystems are poorly understood. Here, seagrass sediments (SS) exhibited significantly (*P* < .05) lower nitrification and N_2_O production rates than non-seagrass sediments, where nitrification was the dominant N_2_O source. We characterized Comammox for the first time in a seagrass ecosystem, revealing that they displayed potential nitrification rates lower than ammonia-oxidizing bacteria or ammonia-oxidizing archaea. Notably, Comammox showed the lowest potential N_2_O production rates and negative CO_2_ fluxes. Metagenomic profiles revealed lower relative abundances of genes associated with nitrification and N_2_O-producing pathways in SS, while metatranscriptomic analysis identified significant (*P* < .05) down-regulation of the core nitrification genes. In contrast, genes involved in sugar transport and metabolism generally showed positive transcriptional changes. This study identifies a key microbial mechanism through which seagrass-derived carbon may contribute to lower nitrification and N_2_O emissions, highlighting the potential of microbiome engineering in seagrass restoration and conservation for climate mitigation.

## Introduction

Nitrous oxide (N_2_O), the third most abundant long-lived greenhouse gas (GHG), possesses a 100-year global warming potential approximately 270 times greater than that of carbon dioxide (CO_2_) [1]. Over the past 150 years, rising atmospheric N_2_O concentrations have exacerbated both stratospheric ozone depletion and climate change [2]. Nitrification and denitrification are the principal biogeochemical processes regulating N_2_O emissions from ecosystems, including coastal wetlands, agricultural soils, and wastewater treatment plants [2]. Both processes are influenced by a suite of environmental factors, including organic carbon (C) substrates, nitrogen (N) availability, and pH. Globally, nitrification is estimated to contribute more to N_2_O emissions than denitrification, accounting for an annual N flux of ~2000 Tg [3]. However, nitrification accounts for 44.3% of N_2_O emissions in wetland ecosystems [3], with abundance of ammonia-oxidizing archaea (AOA) serving as a key regulator that could be amplified by ongoing global warming and environmental changes [4]. Therefore, elucidating nitrifier ecology and N_2_O pathways is critical for predicting emissions and developing mitigation strategies.

Traditionally, nitrification was described as a two-step process catalyzed by distinct microbial guilds: AOA and ammonia-oxidizing bacteria (AOB) oxidize ammonia (NH_3_) to nitrite (NO_2_^−^) via hydroxylamine (NH_2_OH), and nitrite-oxidizing bacteria (NOB) subsequently oxidize nitrite to nitrate (NO_3_^−^) [5]. This process generates energy and reducing equivalents required for cell growth, C assimilation, and the establishment of a proton motive force [6]. Their abundance and community composition are commonly assessed using *amoA* and *nxrB*, which encode subunits of ammonia monooxygenase and nitrite oxidoreductase, respectively [7, 8]. Nitrifier communities can also produce N_2_O through two main routes. First, N₂O can be generated through abiotic reactions involving NH₂OH [9], and through cytochrome P460-mediated NH₂OH oxidation, with cytochrome P460 encoded by *cytL* [10]. Also, nitrifier denitrification involves the reduction of NO₂^−^ to NO by nitrite reductase (Nir), followed by the reduction of NO to N₂O by nitric oxide reductase (Nor), which is primarily associated with AOB and favored under low-oxygen conditions [9–12].

The paradigm of nitrification was redefined with the discovery of complete ammonia oxidizers (Comammox), single microorganisms encoded with *amo*, hydroxylamine oxidoreductase (*hao*) and *nxr* genes that catalyze the full oxidation of NH_3_ to NO_3_^−^ [13]. Comammox belong to sublineage II of *Nitrospira*, which also includes canonical NOB [13]. Unlike canonical AOB capable of nitrifier denitrification, the Comammox isolate *Nitrospira inopinata* cannot perform nitrifier denitrification. Instead, its N_2_O production stems exclusively from the abiotic conversion of NH_2_OH, yielding a comparable amount of N_2_O to AOA but much lower than AOB [11]. Therefore, understanding the diversity, function and contribution of Comammox in seagrass ecosystems is important.

Although Comammox are widely distributed across diverse environments, including lakes [14], rivers [15], mangroves [16], riparian zones [17], and salt marshes [18, 19], their diversity, function and ecological contribution in seagrass ecosystems remain unexplored. Seagrass meadows are productive coastal ecosystems, providing C sequestration, nutrient filtration, and biodiversity support [20, 21]. Globally, seagrass meadows contribute to coastal GHG mitigation by sequestering ~192 Tg CO₂ year^−1^, and taking up a net estimated 4.6 Gg N₂O year^−1^ [22]. During growth seasons, decomposing seagrass detritus provides a source of particulate organic C [23, 24]. Crucially, seagrasses exude large quantities of sugars into the rhizosphere, creating localized hotspots with concentrations up to 80-fold higher than typical marine levels [25]. This influx of labile C creates a unique niche that may be exploited by nitrifiers capable of mixotrophic metabolism, allowing them to supplement their autotrophic lifestyle [26–29]. However, this environment is further complicated by the release of phenolic compounds, which can inhibit microbial activity [25] and intensify competition between the seagrass and microorganisms for N sources [30, 31]. Therefore, the high-C, nutrient-competitive, and potentially inhibitory conditions in the seagrass rhizosphere impose strong selective pressures on sediment microbial communities, particularly for slow-growing, sensitive microorganisms like nitrifiers, which must adapt to capitalize on available organic C to survive this complex niche [5, 32, 33].

The effects of these pressures on nitrification and N_2_O fluxes remain unclear with conflicting reports in the literature. For instance, Lin and colleagues reported higher potential nitrification rates in *Zostera marina* sediments compared to bare sediments, despite a lower nitrifier abundance [34]. In contrast, another study observed lower N_2_O fluxes from seagrass (*Z. marina* and *Zostera japonica*) meadows than from bare sediments, suggesting that heterotrophic processes might instead dominate N_2_O production [35]. Consequently, considerable uncertainty persists regarding the activity of canonical nitrifiers in seagrass meadows, while the contribution of Comammox and their N_2_O production mechanisms are completely unknown. Resolving these uncertainties is crucial for accurately assessing the role of seagrass meadows in mitigating climate change.

*Halophila ovalis* is a pioneer seagrass commonly found in dynamic and disturbed environments [36]. In Hong Kong, it is widely distributed and forms mature meadows in the Pearl River Estuary, a nutrient-rich system subject to seasonal freshwater discharge and terrestrial inputs [37, 38]. The *H. ovalis* ecosystem therefore serves as a critical C accumulator by producing and accumulating C to support high sediment C stocks that drive biogeochemical cycling [38]. In this study, we investigated how *H. ovalis* presence influences the structure, function, and ecological contribution of nitrifier communities. We hypothesized that seagrass-derived organic C inputs would increase organic C utilization by nitrifiers while reducing nitrification and nitrifier-driven N₂O production in seagrass sediments (SS) relative to non-seagrass sediments (NS). Accordingly, SS were expected to show lower abundance and transcription of nitrification-related genes and higher abundances and transcript levels of genes involved in sugar transport and organic C metabolism. To test this hypothesis, microcosms and integrated molecular analyses (e.g. qPCR, metagenomics, metatranscriptomics) were conducted to dissect the diversity, expression of key genes, and function of nitrifiers. Potential nitrification rates and nitrifier-associated N₂O production rates were lower in SS than in NS. This work elucidates how seagrasses regulate N cycling and GHG fluxes via their influence on the nitrifier communities, providing novel insights for the conservation and restoration of these vital coastal blue C ecosystems.

## Materials and methods

### Study site and sample collection

In April 2024, sediments were collected from an intertidal *H. ovalis* seagrass meadow at San Tau Pier, Tung Chung Bay, Lantau Island, Hong Kong (22°17′14″ N, 113°55′31″ E; Fig. S1). A total of 26 topsoil samples (0–9 cm) from SS and adjacent NS were collected for metagenomic analysis, and 18 of these were also used for microbial activity assays, physicochemical analysis, and metatranscriptomic analysis. Each sample was homogenized and partitioned, with subsamples for microbial activity stored at 4°C and those for molecular analyses stored at −80°C.

### Physicochemical properties assay

Temperature and pH were measured in situ with an electronic probe (Hydrolab MS5, USA) [39]. Salinity was measured using a salinity meter (EUTECH SALT6+, Thermo Scientific, USA). TOC was measured using a TOC analyzer (Shimadzu TOC-L CPH/CPN, Japan). NH_4_^+^, NO_3_^−^ and NO_2_^−^ were extracted with 2 M KCl and measured using a multimode microplate reader (Varioskan LUX, Thermo Scientific, USA). Porewater for silicate, phosphate, and TON was analysed via a continuous flow analyzer (SEAL AA500, Seal Analytical, Mequon, USA).

### Potential nitrification and N_2_O production rates

Activity of nitrifiers was determined using a triple-inhibitor microcosm assay with potassium chlorate (KClO_3_) [40], 1-octyne [41], and tetramethylimidazoline-1-oxide-3-oxyl (PTIO) [42]. Briefly, 5.0 g of homogenized sediment was placed into 60 ml serum bottles, sealed with butyl rubber stoppers and aluminum caps, and pre-incubated at 25°C for 2 days to stabilize microbial activity. Four treatment combinations were established: (i) control without inhibitors; (ii) 1 mM KClO₃; (iii) 1 mM KClO₃ + 10 μM 1-octyne; and (iv) 1 mM KClO₃ + 10 μM 1-octyne +100 μM PTIO. The microcosms were incubated in the dark at 25°C and destructively sampled on Days 0, 1, 2, and 4 (Details are provided in the Supporting methods and Fig. S2).

### Sediment microbial community DNA extraction and quantitative PCR analysis

Total microbial community DNA was extracted from 0.5 g using a DNeasy PowerSoil Pro Kit (Qiagen, Germany). The quality and quantity of DNA were assessed using a NanoDrop 2000 spectrophotometer (Thermo Fisher Scientific, USA). Nitrifier abundances were quantified via quantitative PCR (qPCR) using the FastStart Universal SYBR Green Master (Rox) mix (Roche, Germany). Specificity was confirmed by melt curve analysis. All samples and standards were run in triplicate, and only assays with amplification efficiencies of 90%–110% and R^2^ > 0.99 were accepted. Primer and thermocycling protocols are in Table S1.

### Amplicon sequencing and data analysis

Functional genes were amplified using the primer sets and thermocycling protocols detailed in Table S2. PCR products were purified using AMPure PB beads (Pacific Biosciences, USA) and quantified using a Qubit 4.0 fluorometer (Thermo Fisher Scientific, USA). Purified amplicons were sequenced on an Illumina NextSeq 2000 platform (Illumina, USA). The resulting sequences were then quality-filtered using fastp (v0.19.6) [43], and paired-end reads were merged using FLASH (v1.2.11) [44]. High-quality merged sequences were processed with DADA2 [45] in QIIME 2 (v2020.2) [46]. Amplicon datasets for comammox *amoA*, AOA *amoA*, AOB *amoA*, and *Nitrospira nxrB* were analysed separately; comammox-specific patterns were inferred only from comammox *amoA*.

### Metagenome sequencing and data analysis

Metagenome libraries were sequenced on a NovaSeq 6000 (Illumina, San Diego, CA, USA). Raw reads were processed with Trimmomatic (v0.39) [47] and assessed using FastQC (v0.12.1) [48]. Cleaned reads were co-assembled using MEGAHIT (v1.2.9) [49] and classified with Kraken2 (v2.1.2) [50].

Open reading frames were predicted using Prodigal (v2.6.3) [51] and a non-redundant gene catalog was generated using CD-HIT (v4.8.1) [52]. Gene abundance was quantified using Salmon (v1.9.0) [53]. Function was annotated using DIAMOND (v2.0.15) [54] against NCycDB (v2021) [55] and KEGG [56]. MAGs were recovered from MetaBAT2 (v2.15.3) [57], MaxBin2 (v2.2.7) [58], and CONCOCT (v1.1.0) [59], refined using MetaWRAP (v1.3) [60], assessed with CheckM (v1.1.2) [61], and classified with GTDB-Tk (v1.6.0) [62]. MAGs were annotated using Prokka (v1.13) [63], and metabolic pathways were reconstructed using METABOLIC (v4.0) [64], eggNOG (v4.5) [65], dbCAN3 (v14) [66], and KEGG Mapper (v5.2) [56] based on annotations from GhostKOALA [67].

### Sediment microbial community RNA extraction and metatranscriptome sequencing analysis

RNA was extracted using RNeasy PowerSoil Total RNA Kit (Qiagen, Germany) and assessed with NanoDrop spectrophotometer. Following rRNA depletion using the Ribo-Zero Plus rRNA Depletion Kit (Illumina), metatranscriptome libraries were prepared and sequenced on an Illumina NovaSeq 6000. Raw sequence quality was evaluated with FastQC (v0.12.1) [48], and reads were trimmed with Trimmomatic (v0.39) [47]. Remaining rRNA sequences were removed using SortMeRNA (v4.3.7) [68]. Transcript abundance was quantified using Salmon (v1.9.0) [53]. Differential expression analysis was performed using DESeq2. Genes with |log₂FC| ≥ 1 and Benjamini–Hochberg-adjusted *P* value (FDR) < .05 were defined as significantly differentially expressed.

### Statistical analysis

Alpha-diversity and beta-diversity were calculated using QIIME 2. Differences in potential nitrification, N₂O flux, and CO₂ flux between paired SS and NS were tested using two-sided paired t-tests based on replicate-level derived rates. Analysis of similarities (ANOSIM) and permutational multivariate analysis of variance (PERMANOVA; Adonis) were performed based on Bray–Curtis dissimilarity matrices with 999 permutations to test differences in the nitrifier community composition between SS and NS. Environmental variables were Z-score standardized. Pearson’s correlations were calculated among environmental variables, whereas Mantel tests were used to assess associations between environmental variables and nitrifier community composition using the R packages *vegan* and *ggcor*.

## Results

### The activity and contribution of nitrifiers to nitrification and greenhouse gas fluxes

**Figure 1:**
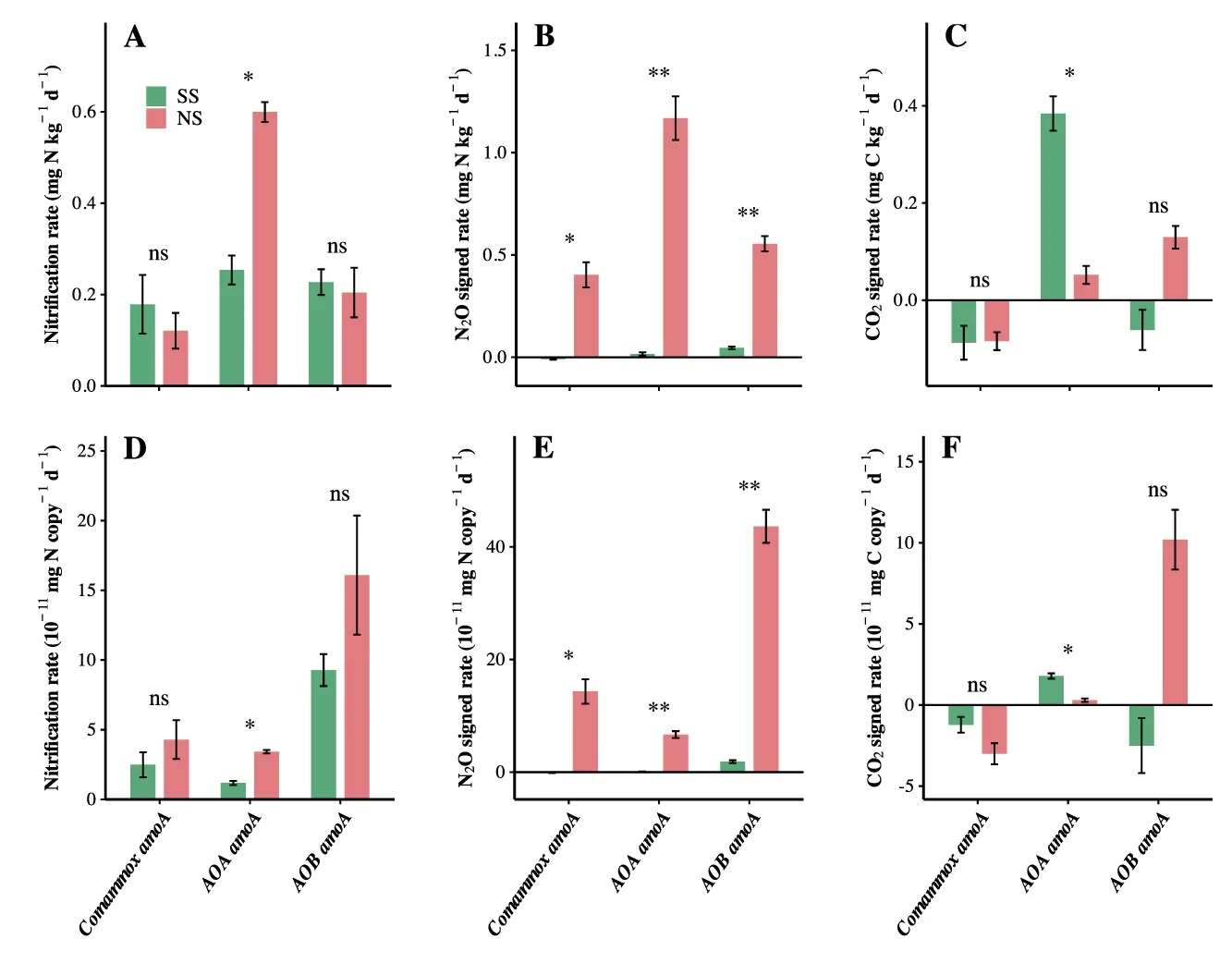
Potential rates of nitrification and associated GHG fluxes. (A–C) Total potential rates of ammonia oxidation, N₂O flux, and CO₂ flux attributed to Comammox, AOA, and AOB in SS and NS sediments. (D–F) The same rates normalized to *amoA* gene copy numbers. Positive values for CO₂ flux indicate net production, while negative values indicate net CO₂ uptake. Data are presented as means ± standard error. Differences between paired SS and NS samples were tested using two-sided paired t-tests based on replicate-level derived rates; asterisks denote significance (^*^P < .05, ^**^P < .01, ^***^P < .001), and NS indicates not significant.

Triple-inhibitor microcosms showed that total potential ammonia oxidation rates were significantly higher in NS (0.939 ± 0.020 mg N kg^−1^ d^−1^) than in SS (0.665 ± 0.030 mg N kg^−1^ d^−1^) (*P* < .05; Fig. S3; Table S3). Potential nitrite oxidation rate was also higher in NS (0.343 ± 0.059 mg N kg^−1^ d^−1^) than in SS (0.214 ± 0.040 mg N kg^−1^ d^−1^) (Fig. S3; Table S3). Comammox exhibited lower ammonia oxidation rates than AOA or AOB (Fig. 1A; Fig. S4A; Table S3). To account for differences in nitrifier abundance, potential rates were normalized to the *amoA* copy numbers of the corresponding groups (Table S4) [11, 69]. After normalization to *amoA* gene copy number, AOB displayed the highest *amoA*-copy-normalized ammonia oxidation rates in both SS and NS, followed by Comammox and AOA (Fig. 1D and Table S3).

We used triple-inhibitor approaches to “quantify the nitrifier-driven N_2_O production” [40, 70–72] “and found that” nitrifier-driven N_2_O production was dramatically suppressed in SS. The nitrifier-associated N₂O flux was approximately 40.9 times higher in NS (2.126 ± 0.135 mg N kg^−1^ d^−1^) than in SS (0.052 ± 0.001 mg N kg^−1^ d^−1^; Fig. S4B; Fig. S5A; Table S5). AOB contributed most in SS, whereas AOA contributed most in NS (Fig. 1B; Table S5). After normalization to *amoA* gene copy number, AOB had the highest N₂O yield in both sediment types, followed by AOA and Comammox (Fig. 1E; Tables S4 and S5).

Nitrifier-associated CO₂ flux differed among groups, with total CO₂ production being 2.42-fold higher in SS (0.235 ± 0.029 mg C kg^−1^ d^−1^) than in NS (0.097 ± 0.012 mg C kg^−1^ d^−1^) (Fig. 1C; Fig. S4C; Fig. S5B; Table S5). AOA and AOB contributed most in SS and NS, respectively, while Comammox-associated CO₂ flux was negative in both sediments. When normalized to *amoA* gene copy number, AOA had the highest positive CO₂-associated rate in SS, whereas AOB had the highest rate in NS. The Comammox-associated rate was negative in both sediment types, and the AOB-associated rate was also negative in SS (Fig. 1F; Table S4 and S5). Overall, SS showed lower potential nitrification and nitrifier-associated N₂O flux but higher AOA-associated CO₂ flux.

### The diversity and composition of nitrifier communities in seagrass sediments

**Figure 2:**
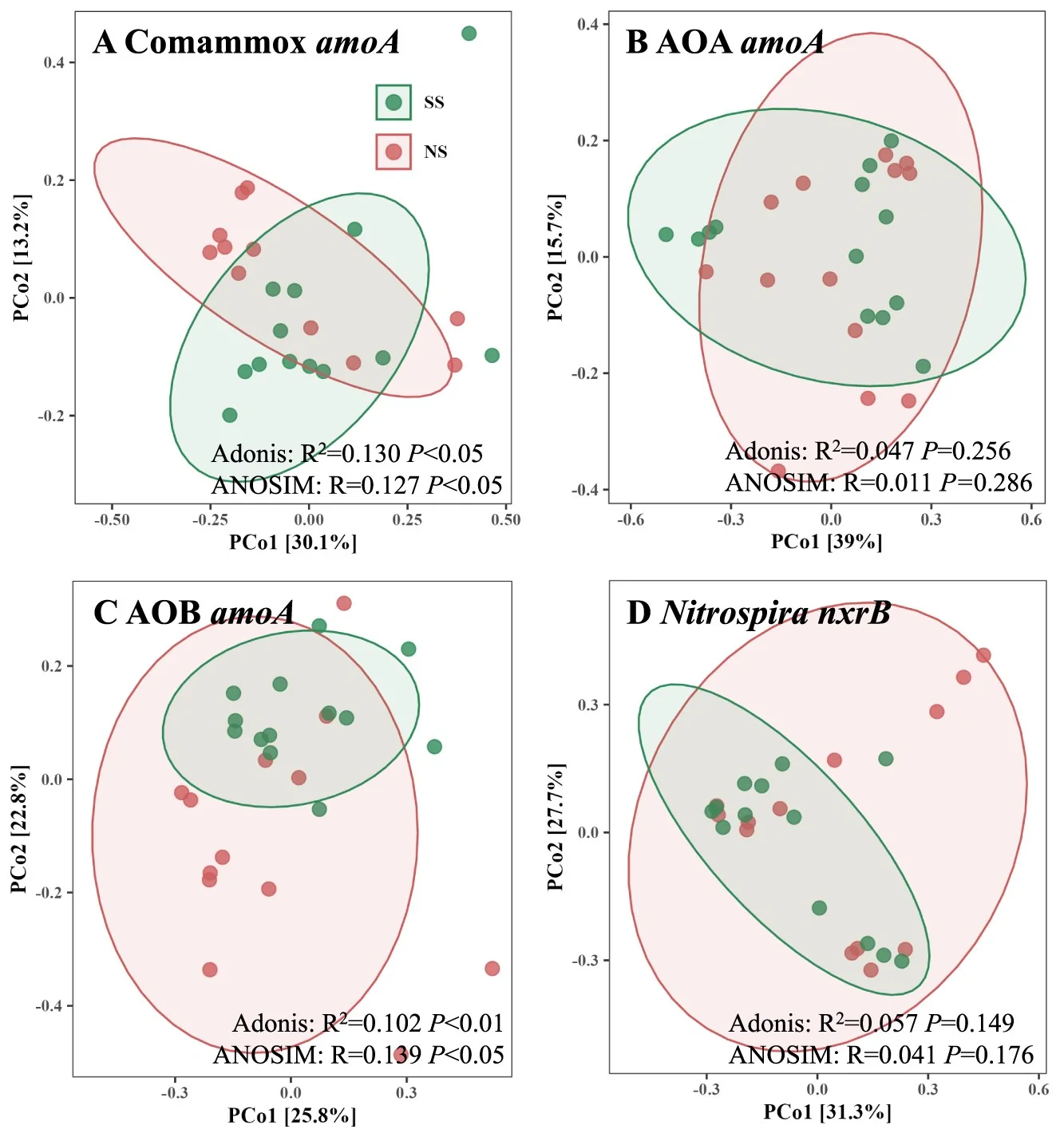
Beta-diversity of nitrifier communities. PCoA based on the Bray–Curtis dissimilarity of ASVs for (A) Comammox *amoA*, (B) AOA *amoA*, (C) AOB *amoA*, and (D) *Nitrospira nxrB* communities in SS and NS. Ellipses represent the 95% confidence interval for each group. The percentage of variation explained by each principal coordinate axis is shown in parentheses. The significance of community structure differences between sediment types was tested using PERMANOVA (Adonis) and ANOSIM.

A total of 807 552, 524 940, 468 960, and 361 416 sequences for Comammox *amoA*, AOA *amoA*, AOB *amoA*, and *Nitrospira nxrB* were obtained, respectively. The AOA communities exhibited the highest alpha-diversity, followed by Comammox and AOB communities (Fig. S6). SS was associated with a reduction in alpha-diversity across all nitrifier communities with statistical significance (*P* < .05) for Comammox and AOA communities. PCoA analysis revealed distinct clustering patterns for the nitrifier communities based on the Bray–Curtis dissimilarity (Fig. 2). The Comammox and AOB community structures differed significantly between SS and NS (Adonis, *P* < .05; ANOSIM, *P* < .05; Fig. 2A and C). In contrast, AOA *amoA* (Adonis, *P* = .256; ANOSIM, *P* = .286; Fig. 2B) and *Nitrospira nxrB* communities did not differ significantly between the two sediment types (Adonis, *P* = .149; ANOSIM, *P* = .176; Fig. 2D). These results indicate that seagrass presence reduced nitrifier diversity and reshaped the structure of Comammox and AOB communities, whereas the structure of AOA and NOB communities remained relatively stable.

### Metabolic characteristics of nitrifiers in seagrass sediments

At the metagenomic genus level, *Nitrosopumilus* (AOA), *Nitrospira*, and *Nitrosomonas* (AOB) were the three most abundant nitrifier genera detected across both sediment types. *Nitrosopumilus* accounted for 0.83% and 1.15% of the total community in SS and NS, respectively; *Nitrospira* represented 0.16% and 0.14%, and *Nitrosomonas* represented 0.09% and 0.10% (Fig. S7A). Within nitrifier communities, the corresponding proportions in SS and NS were 60.30% and 66.75% for *Nitrosopumilus*, 11.59% and 8.10% for *Nitrospira*, and 6.67% and 6.09% for *Nitrosomonas* (Fig. S7B). Because genus-level *Nitrospira* assignments cannot distinguish NOB from Comammox *Nitrospira*, we treated *Nitrospira* as a genus-level taxonomic signal in the metagenomic and metatranscriptomic analyses; Comammox-specific community patterns were assessed from Comammox *amoA* amplicons.

**Figure 3:**
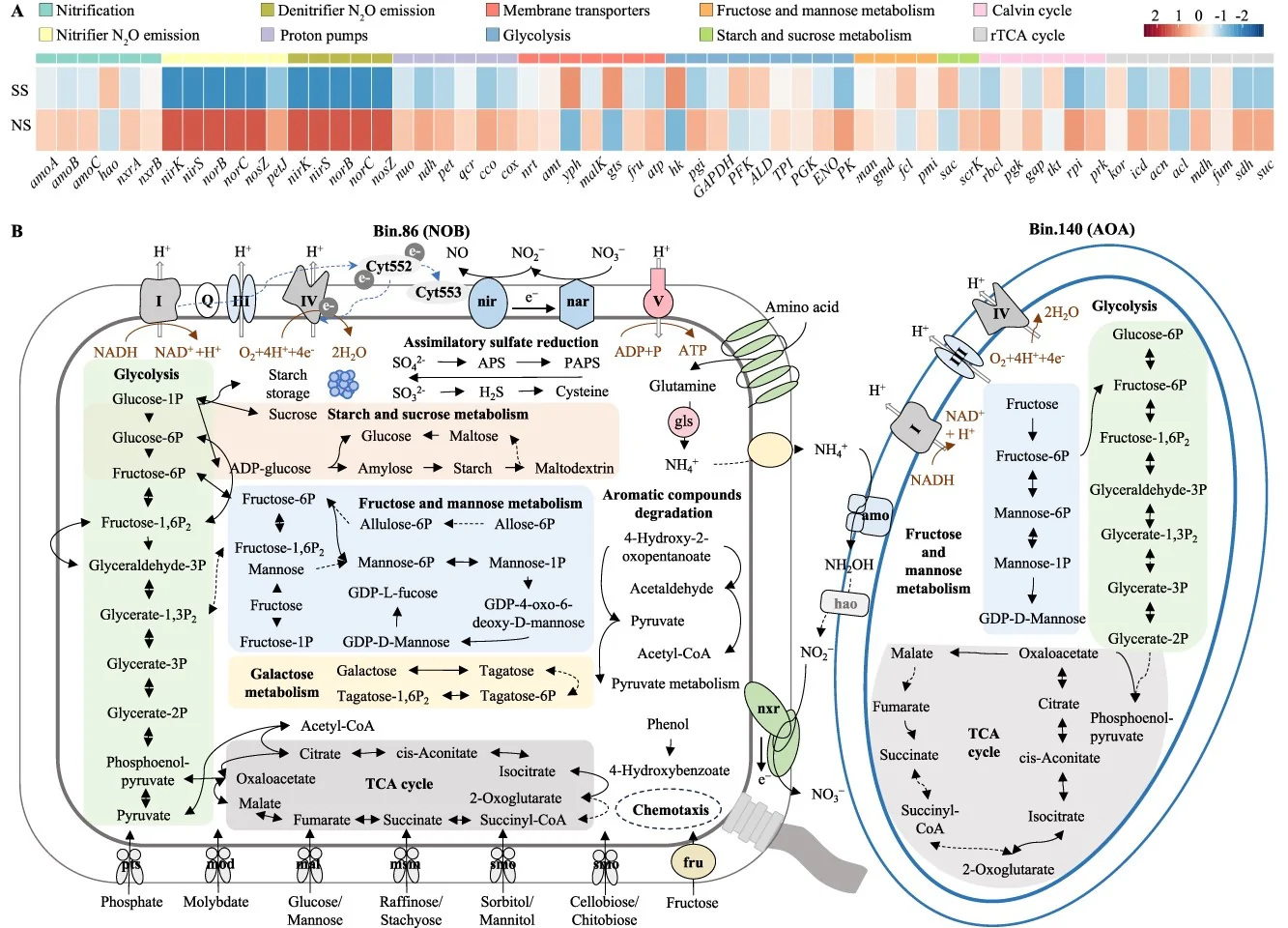
Genomic potential of nitrifier communities. (A) Relationships between gene abundances and sediment types. (B) Reconstructed metabolic pathways of recovered nitrifier MAGs. Metabolic potential of the putative NOB MAG bin.86 (left) and the AOA MAG bin.140 (right). Key metabolic pathways are highlighted in bold. Genes identified within the MAGs are shown in black text, while undetected genes are shown in grey text with dashed lines indicating an incomplete pathway. A full list of gene abbreviations is provided in Table S8.

At the functional gene level, metagenomic profiles revealed a lower overall genetic potential for nitrification and N₂O production in SS. This pattern was reflected in the lower relative abundances of amo genes and nitrite-oxidation genes in SS than in NS (Fig. 3A). Similarly, genes associated with N_2_O production that were assigned to nitrifiers (*nirK*, *nirS, norB*, and *norC*) were generally less abundant in SS. In contrast, genes involved in sugar transport, fructose, mannose, starch, and sucrose showed a more complex and differential distribution in SS, with certain genes being specifically enriched. Furthermore, genes encoding energy-generation components, such as proton pumps, were also found to be less abundant in SS than in NS.

Metagenome binning recovered two types of MAGs representing NOB and AOA lineages although no Comammox or AOB MAGs were recovered. One putative NOB MAG (Bin.86) was 3.07 Mb with 90.59% completeness and 3.76% contamination and was classified within the family Nitrospinaceae (Table S6). One putative AOA MAG (Bin.140) was 0.95 Mb with 58.09% completeness and 1.94% contamination and was classified within the family Nitrosopumilaceae. Both MAGs showed slightly higher relative abundance in NS compared to SS. Bin.86 encoded *nxrA* and *nxrB* genes, indicating its potential for nitrite oxidation (Fig. 3). The genome also encoded *nirK*, indicating the potential for dissimilatory nitrite reduction to nitric oxide, a common trait in NOB [73]. Interestingly, bin.86 also possessed a glutaminase gene (*gls*), suggesting the capacity to produce ammonia from L-glutamine [74]. Bin.140 encoded archaeal *amoA*, supporting ammonia oxidation potential (Table S6).

Both MAGs revealed extensive metabolic capabilities beyond nitrification. Bin.86 encoded a complete electron transport chain, including a terminal oxidase for aerobic respiration (Fig. 3). Both genomes encoded an F-type ATP synthase, cytochrome bc₁ complex, and NADH dehydrogenase, indicating genomic potential for proton-motive-force-dependent ATP synthesis and electron transport. The presence of *nxr* and NADH dehydrogenase genes suggests potential flexibility in electron transfer.

Bin.86 possessed an array of genes and pathways for C utilization. First, a nearly complete Embden–Meyerhof–Parnas pathway for glycolysis and a full tricarboxylic acid (TCA) cycle was detected along with pathways for starch, sucrose, fructose, mannose, and galactose metabolism (Fig. 3). Second, genes encoding glucose-1-phosphate adenylyltransferase, 1,4-alpha-glucan branching enzyme, alpha-amylase, starch synthase, and starch phosphorylase indicated genomic potential for starch metabolism in bin.86. Third, bin.86 encoded a diverse array of carbohydrate-active enzymes for the degradation of complex substrates, including sucrose, starch, xylan, trehalose, polysaccharides, chitin, glycogen, raffinose, and peptidoglycan; the presence of genes for ATP-citrate lyase (*acl*) and pyruvate ferredoxin oxidoreductase (*por*) suggests the capacity for autotrophic C fixation via the reductive TCA (rTCA). Fourth, in agreement with its broad degradation capabilities, bin.86 encoded multiple sugar (e.g. glucose, mannose, fructose) transporters, including several phosphotransferase systems, substrate-specific components (EIIA, EIIB, and EIIC), and ABC-type transporters (*AfuAB*). Fifth, the bin.86 genome contained pathways for the degradation of aromatic compounds, including the conversion of phenol to 4-hydroxybenzoate, as well as the conversion of 4-hydroxy-2-oxopentanoate to acetaldehyde and pyruvate. Also, bin.86 possessed genes for assimilatory sulfate reduction, enabling it to convert SO₄^2−^/SO₃^2−^ to cysteine for biosynthetic needs. Additionally, despite its incompleteness, bin.140 encoded genes for several central C metabolism pathways, including glycolysis, starch and sucrose metabolism, fructose and mannose metabolism, galactose metabolism, and TCA cycle. Collectively, our genomic analysis of the recovered NOB and AOA MAGs revealed that these lineages encoded extensive pathways for C transport and metabolism. Concurrently, community-level metabolic pathways related to sugar transport and degradation were enriched, whereas nitrification and N₂O production gene abundances were reduced in the seagrass environment. These patterns suggest that some nitrifier lineages, particularly the recovered NOB and AOA, possess genetic potentials for mixotrophic metabolism.

### Transcriptional characteristics of nitrifiers in seagrass sediments

Metatranscriptome sequencing further characterized active nitrification and N_2_O production genes/pathways of nitrifier communities. *Nitrosopumilus* (AOA) was the most transcriptionally active nitrifier, comprising 0.78% of total community transcripts in SS and 1.67% in NS (Fig. S7C). Among active nitrifiers, *Nitrosopumilus* transcripts were less dominant in SS (57.72%) than in NS (67.73%), followed by the NOB genus *Nitrospina* (SS: 10.17%; NS: 11.47%), while *Nitrospira* transcripts showed the opposite trend (SS: 11.40%; NS: 6.54%) (Fig. S7D). *Nitrosomonas* was the most active AOB, accounting for 5.0% of nitrifier transcripts in SS and 6.5% in NS, respectively.

**Figure 4:**
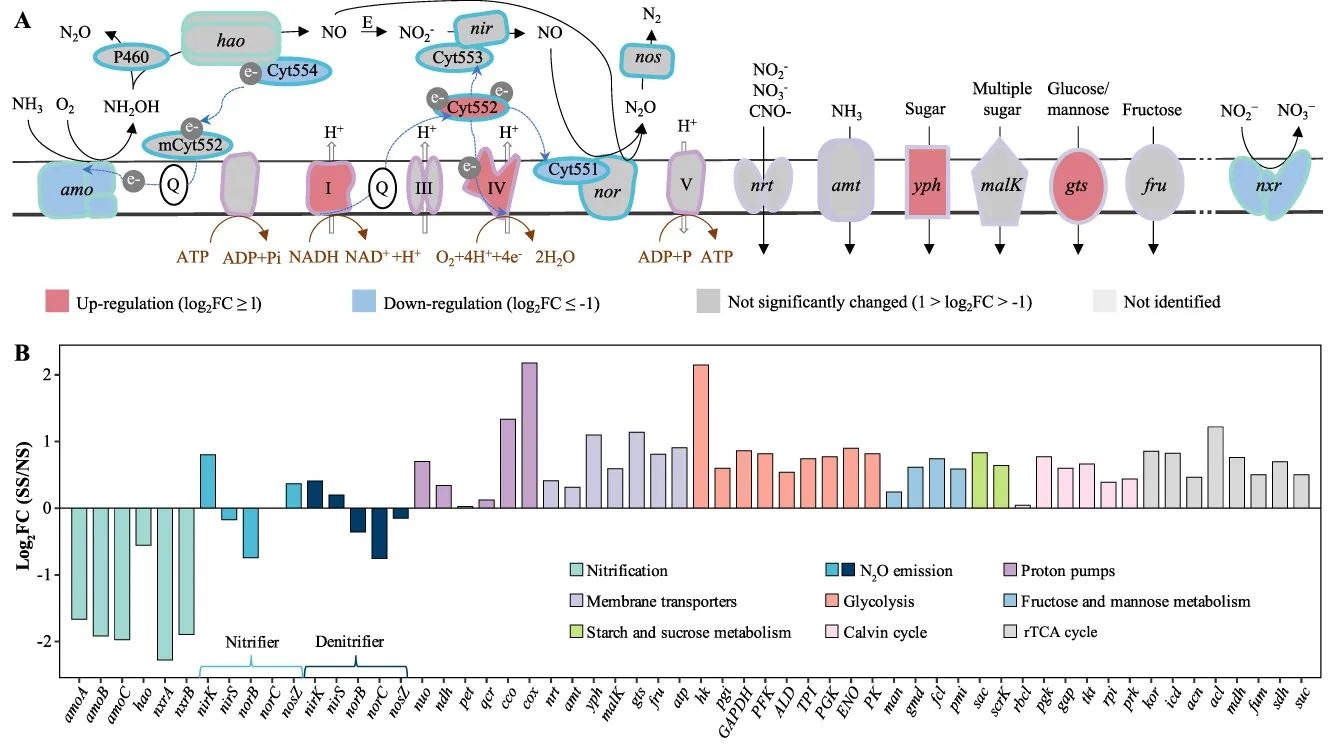
Transcriptional responses of microbial communities to the seagrass environment. (A) A model summarizing the differential expression of genes involved in N and C metabolism. The color of each protein pictogram indicates the log₂-fold change (log₂FC) of its corresponding transcript in SS versus NS. Blue arrows indicate electron flow. (B) Bar chart quantifying the log₂FC for key genes depicted in panel (A). A full list of gene abbreviations is provided in Table S8.

SS showed coordinated down-regulation of core nitrification genes, coinciding with lower potential nitrification and N_₂_O emission rates. Differential expression analysis identified significant down-regulation of *amoA*, *amoB*, *amoC*, *nxrA*, and *nxrB* in SS (log₂FC = −1.67, −1.91, −1.97, −2.28, and − 1.89, respectively; Fig. 4). This transcriptional pattern was consistent with the lower potential nitrification rates measured in SS (Fig. 1A). Genes associated with nitrite and nitric oxide reduction, including *norB*, *nirK*, *nirS*, and *norC*, also showed negative changes, consistent in direction with the lower N_₂_O emissions in SS (Fig. 1B).

In contrast to nitrification-related genes, transcripts associated with sugar transport and metabolism generally showed positive changes in SS. Among the transporter genes, yph and gts, associated with general-sugar and glucose/mannose transport, respectively, were significantly up-regulated (log₂FC = 1.10 and 1.14, respectively; Fig. 4). Genes involved in autotrophic CO₂ fixation through the Calvin cycle and the rTCA cycle also generally showed positive log₂FC values of 0.04–0.77 and 0.46–1.22, respectively. The fructofuranosidase gene *sac*, which is associated with intracellular sucrose utilization, showed a positive 1.78-fold change in SS (log₂FC = 0.83). Transcripts associated with the electron transport chain also showed predominantly positive changes, including *nuo*, *ndh*, *pet*, *qcr*, *cco*, *cox*, and ATP synthase genes, with *cco* and *cox* showing the largest increases (log₂FC = 1.33 and 2.18, respectively; Fig. 4). These transcriptional profiles were consistent with the metagenomic patterns that the seagrass environment repressed nitrification and N_2_O production pathways, while C transport and metabolism showed an increasing trend, providing transcriptional evidence for potential shifts toward organic-carbon acquisition in “specific nitrifier communities”*.*

### The relationships between nitrifier communities and physicochemical properties

**Figure 5:**
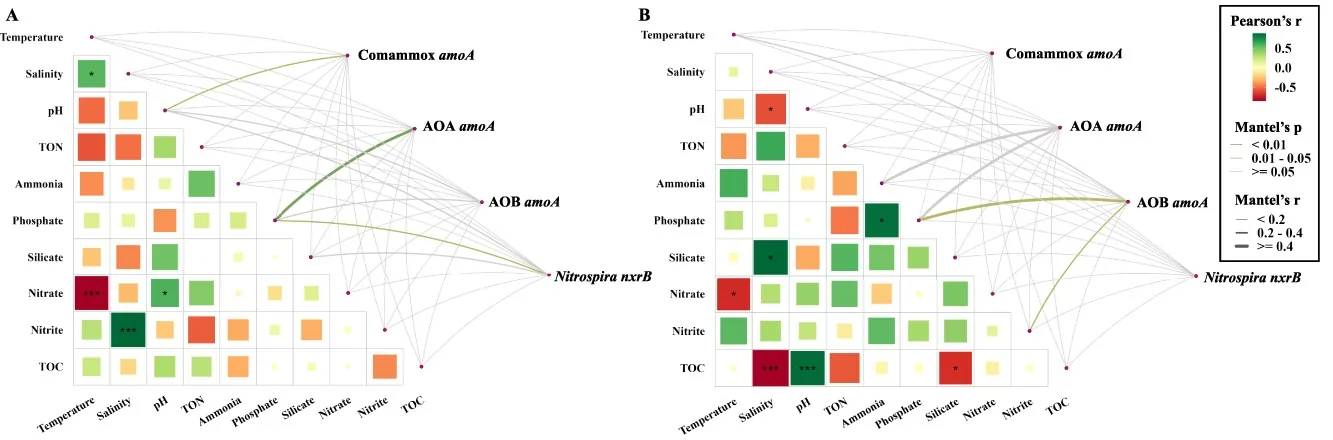
Correlations between nitrifier communities and environmental variables. Mantel tests showing the correlation between the community structure of each nitrifier functional guild and key physicochemical parameters in (A) SS and (B) NS. Matrix colors indicate Pearson’s *r*; network edge width and color indicate Mantel’s *r* and *p*, respectively.

Mantel tests revealed a distinct biogeochemical signature in SS (Table S7). Six of nine parameters differed significantly between SS and NS. Specifically, SS had significantly (*P* < .05) higher concentrations of ammonia, phosphate, salinity, and nitrite, but lower nitrate and pH than NS. Concentrations of TON, silicate, and TOC did not differ significantly (*P* > .05) between SS and NS. In SS, the Comammox community structure was significantly (*P* < .05) correlated with pH, while AOA *amoA* and *Nitrospira* communities were significantly (*P* < .05) correlated with phosphate (Fig. 5A). In NS, phosphate and nitrite were the primary drivers, showing a significant (*P* < .05) association with the AOB *amoA* community structure (Fig. 5B). These results suggest that pH was an important environmental driver for the Comammox community in SS, while nutrient availability (e.g. phosphate, nitrite) primarily affected AOA and *Nitrospira* communities.

## Discussion

Seagrass meadows are important blue C ecosystems where the regulation of nitrification and N_2_O production is governed by a complex interplay of physical and biogeochemical factors. However, the specific microbial mechanisms by which seagrass-derived sugars modulate these transformations have remained elusive. In this study, we aimed to understand how the presence of *H. ovalis* influences the composition, function and N_2_O production of nitrifiers in seagrass ecosystems. By integrating microcosm experiments with multi-omics analyses, we provided the first characterization of Comammox communities in seagrass sediments, and found that nitrifier communities may have shifted their metabolism towards mixotrophy through the enrichment and active transcription of “sugar transport and degradation genes and repressing nitrification and N_2_O production genes/pathways”*.* This trade-off suggests that high availability of root exudates may promote nitrifier communities towards a mixotrophic metabolism, ultimately suppressing autotrophic nitrification and its associated N_2_O flux. These results supported our hypothesis that seagrass-derived organic C would drive the metabolism of sediment nitrifier communities towards mixotrophy, thereby mitigating GHG emissions.

Seagrass-derived organic C may drive a metabolic shift in nitrifier communities, favoring mixotrophic metabolism over energetically costly autotrophy. The seagrass rhizosphere is a C-rich microenvironment abundant in sugars exuded by roots [25], creating a niche that benefits nitrifiers capable of mixotrophic metabolism [28, 75]. “Previous studies” have documented this metabolic flexibility across diverse nitrifier lineages, including comammox *Nitrospira*, AOA, canonical NOB, and some AOB lineages, with varying genomic potentials for organic substrate utilization and central C metabolism. The recovered NOB and AOA MAGs further encoded multiple sugar transporters and organic C utilization pathways, enabling the degradation of complex C compounds and the generation of essential biochemical intermediates [76], and thus supporting similar metabolic potential in the dominant recovered nitrifier lineages. In agreement with these genomic potentials, we also observed enrichment and increased trend of genes involved in sugar transport and degradation, concomitant with down-regulation of core nitrification genes in seagrass sediments. Furthermore, synchronized upregulation of the electron transfer genes and ATP synthesis genes indicated active mixotrophic respiration rather than passive substrate accumulation; physiologically, AOA maintained high CO₂ production rates despite low nitrification rates, suggesting they could actively respire organic C. In contrast, obligate autotrophs constrained to the Calvin cycle, such as specific AOB lineages [77], lacked this metabolic plasticity, thus their CO₂ production was suppressed. For Comammox, the inhibitor-inferred CO₂ flux was negative, suggesting that net CO₂ uptake exceeded CO₂ release, which is consistent with reported rTCA-based autotrophic CO₂ fixation and central C metabolic potential in comammox *Nitrospira* [14, 78]. However, CO₂ is not a pathway-specific nitrification product, and KClO₃ inhibits both Comammox and canonical NOB, thus this signal cannot be unequivocally attributed to Comammox alone. Given that canonical *Nitrospira*/NOB also possess rTCA-based CO₂ fixation and metabolic flexibility [79], we interpret the negative CO₂ flux as an inhibitor-inferred Comammox-associated signal, i.e. consistent with potential autotrophic CO₂ uptake, while acknowledging that contributions from canonical *Nitrospira* cannot be fully excluded. These patterns suggest that seagrass-derived organic C may shift some nitrifiers away from energy-intensive autotrophic nitrification toward more energetically favorable mixotrophic metabolism, allowing them to use organic C while reducing overall nitrification activity and associated N₂O flux [80, 81]. This study provides integrated experimental and multi-omics evidence that seagrass exudation is consistent with enhanced mixotrophic potential by nitrifying communities, a potential mechanism of seagrass-microbe interactions in seagrass sediment.

This metabolic shift towards mixotrophy is consistent with the suppression of nitrification and N_2_O emissions in seagrass sediments. Nitrification is typically a dominant source of *N_2_O* in unvegetated coastal sediments [3], “yet” its contribution varies considerably with “seagrass species, growth stage, and local physicochemical conditions” [35, 82, 83]*.* In this study, low nitrification and “N_2_O production” rates were observed in seagrass sediments, supported by the low abundance and down-regulation of nitrification and “N_2_O-producing genes”. The reduction in N_2_O emissions may be mechanistically explained by the down-regulation of nitrification genes/pathways, which limits the production of hydroxylamine and nitrite, the requisite precursors for N_2_O generation [12]*.* Also, we found that the relative contribution of nitrification to N_2_O emissions decreased substantially in seagrass-colonized sediments, indicating alternative pathways like denitrification could become a primary N_2_O source [35]. Furthermore, our study is the first to identify the specific contribution of Comammox in seagrass ecosystems, revealing that they occupy a unique functional niche with lowest nitrification activity and, notably, the lowest N_2_O emissions among all nitrifiers. This aligns well with a previous study, showing that Comammox lack the enzymatic machinery for nitrifier denitrification, producing N_2_O solely via low-yield abiotic hybrid formation [11]. Although AOB exhibited the highest N₂O yield per amoA-copy-normalized basis, consistent with capacity to conduct nitrifier denitrification [69, 84]*,* AOA drove the highest absolute N₂O emissions due to their dominance in nitrifier communities. By suppressing the overall nitrification activity of nitrifiers, the seagrass environment effectively establishes a community characterized by inherently low N₂O emissions. Therefore, organic C enrichment did not merely passively inhibit nitrification, but also selectively modulate community structure and metabolism to minimize N₂O fluxes, offering a mechanistic explanation for low N₂O emissions in seagrass ecosystems.

Despite the suppression of their primary energy metabolism, nitrifier communities persist in the seagrass rhizosphere through metabolic cross-feeding and stress adaptation. It is well established that NOB can utilize organic N compounds to regenerate ammonia, supporting AOA in N-limited environments [85–87]. Our NOB MAG encoded a salt-tolerant glutaminase capable of converting glutamine to ammonia [74], potentially sustaining AOA in the competitive rhizosphere. Also, the identification of genes for phenolic degradation and assimilatory sulfate reduction suggests these nitrifiers have evolved specific adaptations to detoxify seagrass metabolites and alleviate sulfur limitation in the rhizosphere [25]. These compensatory mechanisms explain why nitrifier communities can survive and maintain their functions in the organic-rich rhizosphere environment.

Although physicochemical limitations or competition for inorganic N in seagrass sediments may result in the suppression of nitrification and N_2_O emissions, our results indicate that such an observation was primarily driven by C availability. First, high C availability theoretically intensifies competition for inorganic N among seagrass, heterotrophic microorganisms, especially slow-growing autotrophs [88, 89]. However, the relative abundance and activity of most denitrifying genes involved in N_2_O emissions did not differ between seagrass and bare sediments, suggesting that N competition with heterotrophs seemed not the primary mechanism. Second, ammonia limitation potentially explains reduced nitrification and associated N_2_O emissions, while ammonia concentrations were higher in seagrass sediments, ruling out the reason for N limitation. Third, oxygen limitation suppresses nitrification in organic-rich sediments, but seagrass actively transports photosynthetically derived oxygen from leaves to roots, enhancing rhizosphere oxygenation [25]. Fourth, while lower pH in seagrass sediment can inhibit nitrifiers, the marginal pH difference between sediments is likely a byproduct of sugar-driven respiration rather than a primary driver [25]. Regarding C availability, the lack of a significant TOC difference between SS and NS does not exclude the influence of seagrass-derived labile C. Here, C availability refers mainly to labile C that can be readily used by microorganisms, whereas TOC represents the total organic C pool. Because labile C represents only a reactive fraction of bulk TOC, differences in labile C availability may not be reflected by total TOC [90]. Therefore, these observations suggest that labile C inputs contributed to the observed metabolic shift and reduced nitrification and N₂O emissions in seagrass sediments.

**Figure 6:**
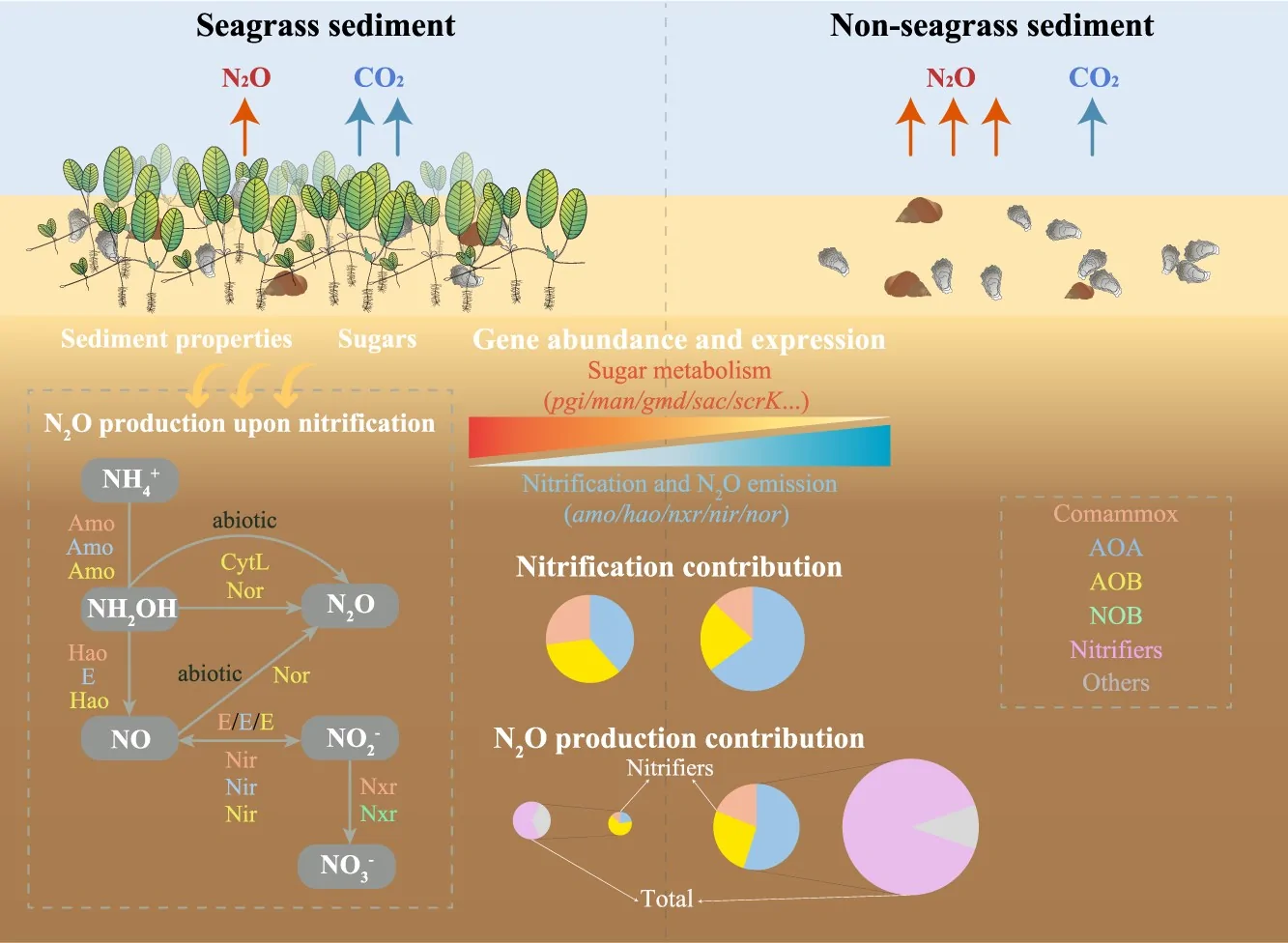
A conceptual model of seagrass-mediated suppression of nitrification and N_2_O emissions in seagrass sediments. The model proposes that labile organic C (e.g. sugars) released by seagrass roots promotes a metabolic shift toward mixotrophy in the benthic nitrifier community. This shift, characterized by increased transcription of C metabolism and reduced transcription of nitrification genes/pathways, is associated with reduced rates of ammonia oxidation and nitrifier-driven N_2_O production. Key enzymatic pathways are shown for Comammox (red), AOA (blue), AOB (yellow), and NOB (green). Definitions of abbreviations used in the figure are provided in Table S8. Organismal icons were obtained and adapted from the University of Maryland Center for Environmental Science’s Integration and Application Network and Flaticon.com; organism sizes are not to scale.

Synthesizing these findings, we developed a conceptual model, in which seagrass-derived labile organic C may contribute to a shift in nitrifier communities toward mixotrophic metabolism, resulting in a coordinated suppression of nitrification activity and associated N_2_O flux in the seagrass sediment (Fig. 6). Such a metabolic shift is evidenced by reduced abundances and down-regulation of nitrification genes/pathways along with increased transcription of C transport and metabolism genes. Future investigations will integrate stable isotope tracing, multi-omics analysis, and experimental validation of reduced nitrification and N_2_O production using synthetic microbial communities and targeted C amendments. This study provides a key microbial mechanism driven by nitrifier communities, by which seagrass meadows modulate GHG fluxes, highlighting the importance of future microbiome engineering in seagrass restoration and conservation for mitigating global climate change.

## Supplementary Material

Supporting_information_ycag220

## Data Availability

Sequence data generated during this study are available in NCBI with accession number PRJNA1436162.
