# Supplementary material for: Seagrass-derived carbon suppresses nitrification and N2O emissions by Comammox and canonical nitrifier communities
Source: ISME Commun. 2026 Jul 29;6(1):ycag220. doi: 10.1093/ismeco/ycag220 (PMC13518056; doi:10.1093/ismeco/ycag220)
Supplement: Supplementary file 1 [file supporting_information_ycag220.docx]

Supporting Information for

**Seagrass-derived carbon suppresses nitrification and N_2_O emissions by Comammox and canonical** **nitrifier communities**

Ruilin Su^1,2^, Pui Ting Chan^1^, Xiaodong Zhang^1^, Jiawei Chen^1^, Sangwook Lee^1^, Xinlei Yang^2^, Hoi Yau Chan^1^, Yuxuan Lin^1^, Jiying Li^1^, Qingyun Yan^2^, Hongbin Liu^1*^, Zhili He^2^^*^

^1^ Department of Ocean Science, Hong Kong University of Science and Technology, Hong Kong 999077, China.

^2^ Marine Synthetic Ecology Research Center, Southern Marine Science and Engineering Guangdong Laboratory (Zhuhai), the China-ASEAN Belt and Road Joint Laboratory on Mariculture Technology, Sun Yat-sen University, Zhuhai 519082, China.

*Address correspondence to: Hongbin Liu, Department of Ocean Science, Hong Kong University of Science and Technology, Clear Water Bay, Kowloon, Hong Kong 999077, China; email: liuhb@ust.hk.

Zhili He, Marine Synthetic Ecology Research Center, Southern Marine Science and Engineering Guangdong Laboratory (Zhuhai), No. 2 University Road, Xiangzhou District, Zhuhai, Guangdong 519082, China; email: hezhili@sml-zhuhai.cn.

A. **Supporting methods**
B. **Supporting figures**

**Fig. S1** Study site overview. (A) Satellite map showing the location of the study site at San Tau Pier, Tung Chung Bay, Lantau Island, Hong Kong (source: Google Earth Pro). Field photographs of the intertidal zone flat show (B) seagrass sediments (SS) and (C) non-seagrass sediments (NS).

**Fig. S2** Schematic of the triple-inhibitor assay. Diagram illustrating the four experimental treatments used to differentiate the activities of AOA, AOB and Comammox.

**Fig. S3** Time-course of NO_x_^-^ (NO_2_^-^ + NO_3_^-^) accumulation over the 4-day incubation period in SS and NS with different inhibitor treatments (TRT).

**Fig. S4** Relative contributions of nitrifier guilds to (A) nitrification, (B) N_2_O production, (C) CO_2_ production of three nitrifier guilds in SS and NS.

**Fig. S5** Changes in headspace concentrations of (A) N₂O and (B) CO₂ over the 4-day incubation period under the four experimental treatments.

**Fig. S6** Alpha-diversity of (A) Comammox *amoA*, (B) AOA *amoA*, (C) AOB *amoA* and (D) *Nitrospira nxrB* communities in SS and NS.

**Fig. S7** Taxonomic profiles of sediment microbial communities. Relative abundance of the top 20 most abundant genera within the (A) total and (B) nitrifier communities based on metagenome sequencing data. Relative transcript abundances of the top 20 active genera within the (C) total and (D) nitrifier communities derived from metatranscriptome sequencing data.

C. **Supporting tables**

**Table S1** Primer sets and thermal cycling conditions for qPCR.

**Table S2** Primer sets and corresponding reaction profiles for PCR.

**Table S3** Treatment-specific regression slopes and derived potential nitrification rates.

**Table S4** Abundances of different nitrifiers and total bacteria in SS and NS.

**Table S5** Treatment-specific regression slopes and derived N₂O and CO₂ fluxes.

**Table S6** Quality metrics and summary statistics for the recovered metagenome-assembled genomes (MAGs) of interest.

**Table S7** Physicochemical properties of sediments.

**Table S8** Definitions of abbreviations used in Figs. 3, 4 and 6.

**A. Supporting methods**

***Measuring complete nitrification activity***

**Principles.** The differential activity of Comammox, ammonia-oxidizing archaea (AOA), and ammonia-oxidizing bacteria (AOB) was assessed using a selective inhibitor assay. This approach used potassium chlorate (KClO_3_) to inhibit nitrite oxidation [1, 2], 1-octyne to inhibit AOB [3, 4], and 2-phenyl-4,4,5,5-tetramethylimidazoline-1-oxyl-3-oxide (PTIO) [5–7], a nitric oxide scavenger, to inhibit AOA. As an analog of nitrate, KClO_3_ inhibits the nitrite oxidoreductase (Nxr) enzyme, thereby blocking the oxidation of nitrite to nitrate catalyzed by both canonical nitrite-oxidizing bacteria (NOB) and Comammox. This inhibition also prevents dissimilatory nitrate reduction processes and denitrification [1, 2, 8, 9]. Canonical ammonia oxidation is differentiated using PTIO, which selectively inhibits AOA by scavenging nitric oxide radicals, and 1-octyne, which irreversibly inhibits the ammonia monooxygenase of AOB [6, 7]. By measuring the accumulation of nitrite (NO_2_^−^), nitrate (NO_3_^−^) and N_2_O across different inhibitor combinations, the activity of each nitrifier guild can be calculated (Fig. S2).

Four treatment combinations were established for each sediment sample: (I) control (no inhibitors); (II) 1 mM KClO_3_ (nitrite oxidation inhibition); (III) 1 mM KClO_3_ + 10 µM 1-octyne (nitrite oxidation and AOB inhibition); and (IV) 1 mM KClO_3_ + 10 µM 1-octyne + 100 µM PTIO (total nitrification inhibition). Microcosms were conducted in the dark at 25 °C and sampled on days 0, 1, 2, and 4. Before destructive sampling, a 5 mL headspace gas sample was collected from each bottle with a syringe and injected into pre-evacuated 10 ml glass vials for N_2_O and CO_2_ analysis. Immediately following gas sampling, nitrite and nitrate were extracted from the sediment by adding 25 mL of 2 M KCl and shaking at 200 rpm for 30 min. N_2_O and CO_2_ concentrations were measured by gas chromatography (Agilent 7890A GC, Agilent) using an electron capture detector for N_2_O, and a thermal conductivity detector for CO_2_.

**Analysis.** The measured aqueous concentrations of nitrite and nitrate (mg L^-1^) were converted to mass-based sediment concentrations (mg kg^-1^) using the following equations: (1-2):

$C_{NO_{2}^{-}}=A_{NO_{2}^{-}}\times({(V}_{KCl}+V_{ddH_{2}O\left( KClO_{3} \right)})\div M_{Sediment})$	(1)

$C_{NO_{3}^{-}}=(A_{NO_{x}^{-}}-A_{NO_{2}^{-}})\times{(V}_{KCl}+V_{ddH_{2}O\left( KClO_{3} \right)})\div M_{Sediment}$	(2)

Where $A_{NO_{x}^{-}}$is the measured aqueous concentration (mg L^-1^), $C_{NO_{x}^{-}}$ is the calculated sediment concentration (mg kg^-1^), $V_{KCl}$ is the volume of KCl extractant, $V_{ddH_{2}O\left( KClO_{3} \right)}$ is the volume of added deionized water or inhibitor solution, and $M_{sediment}$is the fresh weight of the sediment. In this study, $V_{KCl}$, $V_{ddH_{2}O\left( KClO_{3} \right)}$, and $M_{sediment}$ were 25 mL, 1.5 mL and 5 g, respectively. Potential rates were determined from the linear regression of analyte concentrations over the incubation period (Fig. S3 and S5).

**Calculations.** The potential rates of ammonia oxidation and nitrite oxidation were calculated from the differential accumulation of nitrite and nitrate across the four treatments. For each analyte in each treatment, concentrations were fitted against incubation time by linear regression, and the resulting slope was denoted as **(**$K_{Treatment analyte}$**)**. The coefficient of determination (R²) ranged from 0.560 to 0.991 for nitrification-related regression (Fig. S3). Potential N_2_O and CO_2_ production rates were assessed through linear regression of N_2_O and CO_2_ accumulation over time, with R² values of 0.712-0.992 and 0.477-0.985, respectively (Fig. S5). The potential ammonia oxidation rate of AOA was calculated from the NO_2_^-^ accumulation in Treatment III, after correction for NO_2_^-^ accumulation in Treatment IV. Treatment III inhibited AOB and nitrite oxidation activity, whereas Treatment IV inhibited AOA, AOB, and nitrite oxidation (3-4):

$\Delta{NO}_{2_{\left( \mathrm{AOA} \right)}}^{-}=\Delta{NO}_{2(III)}^{-}-\Delta{NO}_{2_{(IV)}}^{-}$	(3)

$K_{\mathrm{AOA}}=K_{{NO}_{2_{(III)}}^{-}}-K_{{NO}_{2_{(IV)}}^{-}}$	(4)

Where $\Delta{NO}_{2_{(\mathrm{III})}}^{-}$and $\Delta{NO}_{2_{(\mathrm{IV})}}^{-}$represent the accumulation of ${NO}_{2}^{-}$ in treatments $\mathrm{III}$ and IV, respectively.$K_{{NO}_{x_{y}}^{-}}$represents the accumulation slope of ${NO}_{x}^{-}$ over time in the treatment *y*. $K_{\mathrm{AOA}} \mathrm{and}\Delta{NO}_{2_{(AOA)}}^{-}$represent the potential archaeal ammonia oxidation activity (AOA activity) and the NO_2_^-^ accumulation derived from AOA, respectively. The potential ammonia oxidation rate of AOB was calculated as the difference between the ${NO}_{2}^{-}$ accumulation rate in treatment II and treatment III. Treatment IV represents NO₂⁻ production by non-nitrifying microorganisms, and its contribution is present in both treatments II and III and thus cancels out when the difference is taken (5-7):

$\Delta{NO}_{2(II)}^{-}= \Delta{NO}_{2_{(AOA)}}^{-}+\Delta{NO}_{2_{(AOB)}}^{-}+\Delta{NO}_{2_{(IV)}}^{-}$	(5)

$\Delta{NO}_{2(III)}^{-}=\Delta{NO}_{2(AOA)}^{-}+\Delta{NO}_{2(IV)}^{-}$	(6)

$K_{\mathrm{AOB}}=K_{{NO}_{2_{(II)}}^{-}}-K_{{NO}_{2_{(III)}}^{-}}$	(7)

Where $\Delta{NO}_{2(II)}^{-}$ represents the accumulation of NO_2_^-^ in treatment II. $K_{\mathrm{AOB}} and \Delta{NO}_{2(AOB)}^{-}$represent the potential bacterial ammonia oxidation activity (AOB activity) and the NO_2_^-^ accumulation from AOB, respectively. The potential nitrite oxidation rate was calculated as the difference between the NO_2_^-^ accumulation rate in treatment II and treatment I (8-10):

$\Delta{NO}_{2(I)}^{-}= \Delta{NO}_{2_{(AOA)}}^{-}+\Delta{NO}_{2_{(AOB)}}^{-}+\Delta{NO}_{2_{(IV)}}^{-}-\Delta{NO}_{2_{\left( Nitrite oxidation \right)}}^{-}$	(8)

combining Equations (5) and (8) yields:

$\Delta{NO}_{2_{\left( Nitrite oxidation \right)}}^{-} =\Delta{NO}_{2_{(II)}}^{-}-\Delta{NO}_{2_{\left( I \right)}}^{-}$	(9)

$K_{Nitrite oxidation}=K_{{NO}_{2_{(II)}}^{-}}-K_{{NO}_{2_{(I)}}^{-}}$	(10)

Where $\Delta{NO}_{2_{\left( I \right)}}^{-}$ represents the accumulation of NO_2_^-^ in treatment I. $K_{Nitrite oxidation}$represents the potential nitrite-oxidizing process activity, i.e., the NO_2_^-^ oxidation activity through canonical nitrification and $\Delta{NO}_{2_{(Nitrite oxidation)}}^{-}$represents the NO_2_^-^ consumption derived from canonical nitrite-oxidizing bacteria. The potential rate of complete ammonia oxidation by Comammox was estimated from total nitrification-derived NO_3_^-^ formation, calculated as the difference between the NO₃⁻ accumulation slopes in treatment I and treatment II (11-12):

$\Delta{NO}_{3_{(Total nitrification)}}^{-}=\Delta{NO}_{3_{(Nitrite oxidation)}}^{-}+\Delta{NO}_{3_{(Comammox)}}^{-}$	(11)

$K_{\mathrm{Comammox}}=K_{\Delta{NO}_{3_{(Total nitrification)}}^{-}}-K_{Nitrite oxidation}$	(12)

Where$K_{\mathrm{Comammox}}$, $\Delta{NO}_{3_{(Nitrite oxidation)}}^{-}$, $\Delta{NO}_{3_{(Comammox)}}^{-}$, and $\Delta{NO}_{3_{(Total nitrification)}}^{-}$ represent the potential Comammox activity, the NO_3_^-^ accumulation derived from nitrite oxidizing process, the NO_3_^-^ accumulation derived from Comammox and the total accumulation of NO_3_^-^, respectively. Compared to the incubations of treatment I and II, the total accumulation activity of NO_3_^-^ was calculated by equation (13):

$K_{\Delta{NO}_{3_{(Total nitrification)}}^{-}}=K_{\Delta{NO}_{3_{(I)}}^{-}}-K_{\Delta{NO}_{3_{(II)}}^{-}}$ (13)

The total potential N_2_O and CO_2_ production rate from nitrifiers was calculated as the difference between the rate in the control (treatment I) and the rate in treatment IV, where all nitrification was inhibited (14-17). For N₂O, the resulting inhibitor-sensitive differences were reported as flux magnitudes, whereas CO₂ differences were retained as signed fluxes; uncertainties were conservatively propagated as the sum of the standard errors of the corresponding treatment-specific slopes.

$K_{{Gas}_{(AOA)}}=K_{\Delta{Gas}_{(III)}}-K_{\Delta{Gas}_{(IV)}}$	(14)

$K_{{Gas}_{(AOB)}}=K_{\Delta{Gas}_{(II)}}-K_{\Delta{Gas}_{(III)}}$	(15)

$K_{{Gas}_{(Comammox)}}=K_{\Delta{Gas}_{(I)}}-K_{\Delta{Gas}_{(II)}}$	(16)

$K_{{Gas}_{(Total microbes)}}=K_{\Delta{Gas}_{(I)}}-K_{\Delta{Gas}_{(IV)}}$	(17)

The relative contributions of ammonia oxidizers to the consumption of NH_4_^+^ or the production of N_2_O and CO_2_ were determined by dividing the potential rates of ammonia oxidizers by the overall NH_3_ oxidation rate or the total N_2_O/CO_2_ production rate associated with NH_3_ oxidation as shown in equation (18).

${{{NH}_{4}}^{+}/N_{2}O/{CO}_{2} contribution}_{(Comammox/AOA/AOB)}=\frac{{Rate}_{(Comammox/AOA/AOB)}}{{Rate}_{(Comammox+AOA+AOB)}}\times100\%$	(18)

The N_2_O and CO_2_ contribution of nitrifiers can be illustrated in the following equation:

${N_{2}O/{CO}_{2} contribution}_{(Nitrifiers)}=\frac{{Rate}_{(Nitrifiers)}}{{Rate}_{(Total microbes)}}\times100\%$	(19)

**B. Supporting figures**

**Fig. S1** Study site overview. (A) Satellite map showing the location of the study site at San Tau Pier, Tung Chung Bay, Lantau Island, Hong Kong (22°17′14″ N, 113°55′31″ E) (source: Google Earth Pro). Field photographs of the intertidal zone flat show (B) seagrass sediments (SS) and (C) non-seagrass sediments (NS).

**Alt text:** Map and field photographs showing the SS and NS sampling sites at San Tau Pier, Tung Chung Bay, Hong Kong.

**Fig. S2** Schematic of the triple-inhibitor assay. Diagram illustrating the four experimental treatments used to differentiate the activities of AOA, AOB and Comammox.

**Alt text:** Schematic diagram showing the four inhibitor treatments used to distinguish AOA, AOB, and Comammox activities.

**Fig. S3** Time-course and linear regression of NO₂⁻ and NO₃⁻ accumulation over the 4-day incubation period in SS and NS under different inhibitor treatments. The four panels show the regression curves used to estimate AOA-, AOB-, Comammox-associated ammonia oxidation and potential nitrite oxidation. Treatment-specific regression slopes and derived potential nitrification rates are provided in Table S3.

**Alt text:** Time-course regression plots showing NO₂⁻ and NO₃⁻ accumulation under different inhibitor treatments in SS and NS.

**Fig. S4** Relative contributions of nitrifiers to (A) nitrification, (B) N_2_O production, (C) CO_2_ production of three nitrifiers in SS and NS. Orange bars, green bars and purple bars represent Comammox, AOB and AOA, respectively. Data are represented as mean ± standard error. Differences between paired SS and NS samples were tested using two-sided paired t-tests based on replicate-level derived rates; asterisks denote significance (**p* < 0.05, ***p* < 0.01, ****p* < 0.001), and ns indicates not significant.

**Alt text:** Bar plots showing the relative contributions of Comammox, AOB, and AOA to nitrification, N₂O flux, and CO₂ flux in SS and NS.

**Fig. S5** Time-course and linear regression of greenhouse gas accumulation. Changes in headspace accumulation of (A) N₂O and (B) CO₂ over the 4-day incubation period under the four inhibitor treatments. Treatment-specific regression slopes and derived N₂O and CO₂ production rates are provided in Table S5.

**Alt text:** Time-course regression plots showing N₂O and CO₂ accumulation under different inhibitor treatments in SS and NS.

**Fig. S6** Alpha-diversity of (A) Comammox *amoA*, (B) AOA *amoA*, (C) AOB *amoA* and (D) *Nitrospira nxrB* communities in SS and NS. Chao index, Shannon index, and Observed ASVs were calculated using rarefied data. Statistical significance was determined by the Kruskal-Wallis test.

**Alt text:** Box plots comparing alpha diversity indices of Comammox *amoA*, AOA *amoA*, AOB *amoA*, and *Nitrospira nxrB* communities between SS and NS.

**Fig. S7** Taxonomic profiles of sediment microbial communities. Relative abundance of the top 20 most abundant genera within the (A) total and (B) nitrifier communities based on metagenome sequencing data. Relative transcript abundances of the top 20 active genera within the (C) total and (D) nitrifier communities derived from metatranscriptome sequencing data.

**Alt text:** Stacked bar plots showing genus-level taxonomic profiles of total and nitrifier-related communities based on metagenomic and metatranscriptomic data.

**C. Supporting tables**

**Table S1** Primer sets and thermal cycling conditions for qPCR.

| Specificity | Primer | Sequence (5’-3’) | Thermal profiles | Reference |
| --- | --- | --- | --- | --- |
| AOA *amo*A | archea-*amo*AF | STAATGGTCTGGCTTAGACG | 5 min at 95 °C, 35 cycles consisting of 45 s at 94 °C, 1 min at 53°C and 1 min at 72°C. | [10] |
|  | archea-*amo*AR | GCGGCCATCCATCTGTATGT |  |  |
| AOB *amo*A | *amo*A-1F | GGGGTTTCTACTGGTGGT | 5 min at 95 °C, 35 cycles consisting of 30 s at 94 °C, 30 s at 55 °C and 1 min at 72 °C. | [11] |
|  | amoA-2R | CCCCTCKGSAAAGCCTTCTTC |  |  |
| Comammox *amo*A | comamoA F | AGGNGAYTGGGAYTTCTGG | 95 °C for 15 min, followed by 40 cycles of denaturation at 95 °C for 30 s, annealing at 53 °C for 30 s and extension at 72 °C for 1 min. | [12] |
|  | comamoA R | CGGACAWABRTGAABCCCAT |  |  |
| *Nitrospira nxrB* | nxrB169F | GTACTGGTGGGCBAAYTT | 95 °C for 5 min followed by 40 cycles of denaturation (95 °C, 40 s), primer annealing at 55 °C, 40 s, and elongation at 72 °C, 90 s, with 10 min of final extension at 72 °C. | [13] |
|  | nxrB638R | GAAGCCCATRTARTCNGCC |  |  |
| 16S | 8F | AGAGTTTGATYMTGGCTCAG | 3 min at 95 °C, 40 cycles consisting of 30 s at 95 °C, 30 s at 55 °C and 30 s at 68 °C | [14] |
|  | Eub338-I R | GCTGCCTCCCGTAGGAGT |  |  |

**Table S2.** Primer sets and corresponding reaction profiles for PCR.

| Specificity | Primer | Sequence (5’-3’) | Thermal profiles | Reference |
| --- | --- | --- | --- | --- |
| AOA *amo*A | AOA83F64 | GGWGAYTAYATNTTCTAYAC | denaturation at 94 °C for 1 min, 30 cycles of 94 °C for 20 s, 57°C for 25 s and 68 °C for 45 s, thereafter extension at 68 °C for 10 min and then held at 4 °C. | [15] |
|  | AOA518R64 | GGRCTRTTRTAGAAYTTNCC |  |  |
| AOB *amo*A | *amoA*-1F | GGGGTTTCTACTGGTGGT | 3 min at 95 °C, 35 cycles of (30 s at 95 °C, 45 s at 56 °C, and 60 s at 72 °C), 5 min at 72 °C. | [11] |
|  | *amoA*-2R | CCCCTCKGSAAAGCCTTCTTC |  |  |
| Comammox *amo*A | Ntsp-*amoA* 162F | GGATTTCTGGNTSGATTGGA | 95 °C for 10 min, followed by 25 cycles of 95 °C for 45 s, 48 °C for 30 s, 72 °C for 45 s and final extension at 72 °C for 7 min. | [16] |
|  | Ntsp-*amoA* 359R | WAGTTNGACCACCASTACCA |  |  |
| *Nitrospira nxrB* | *nxrB*169F | GTACTGGTGGGCBAAYTT | 5 min at 95 °C, 35 cycles consisting of 40 s at 95 °C, 30 s at 56.2 °C and 90 s at 72 °C, 10 min at 72 °C. | [13] |
|  | *nxrB*638R | GAAGCCCATRTARTCNGCC |  |  |

**Table S3.** Treatment-specific regression slopes and derived potential nitrification rates. Derived rates are expressed as mg N kg⁻¹ d⁻¹.

| Sediment | Process | Slope used as minuend | Slope used as subtrahend | Difference equation | Derived rate | SE |
| --- | --- | --- | --- | --- | --- | --- |
| SS | AOA ammonia oxidation | TRT III: 0.266 ± 0.025 | TRT IV: 0.001 ± 0.000 | TRT III − TRT IV | 0.264 | 0.020 |
| NS | AOA ammonia oxidation | TRT III: 0.615 ± 0.019 | TRT IV: 0.030 ± 0.005 | TRT III − TRT IV | 0.585 | 0.020 |
| SS | AOB ammonia oxidation | TRT II: 0.497 ± 0.027 | TRT III: 0.266 ± 0.025 | TRT II − TRT III | 0.232 | 0.020 |
| NS | AOB ammonia oxidation | TRT II: 0.838 ± 0.039 | TRT III: 0.615 ± 0.019 | TRT II − TRT III | 0.223 | 0.040 |
| SS | Potential nitrite oxidation | TRT II: 0.497 ± 0.027 | TRT I: 0.283 ± 0.014 | TRT II − TRT I | 0.214 | 0.040 |
| NS | Potential nitrite oxidation | TRT II: 0.834 ± 0.055 | TRT I: 0.491 ± 0.009 | TRT II − TRT I | 0.343 | 0.060 |
| SS | Total nitrification-derived NO₃⁻ formation | TRT I: 0.660 ± 0.038 | TRT II: 0.278 ± 0.017 | TRT I − TRT II | 0.383 | 0.030 |
| NS | Total nitrification-derived NO₃⁻ formation | TRT I: 0.655 ± 0.017 | TRT II: 0.181 ± 0.009 | TRT I − TRT II | 0.474 | 0.020 |
| SS | Comammox ammonia oxidation | Total NO₃⁻ formation: 0.383 ± 0.033 | Potential nitrite oxidation: 0.214 ± 0.040 | Total NO₃⁻ formation − nitrite oxidation | 0.169 | 0.050 |
| NS | Comammox ammonia oxidation | Total NO₃⁻ formation: 0.474 ± 0.022 | Potential nitrite oxidation: 0.343 ± 0.059 | Total NO₃⁻ formation − nitrite oxidation | 0.131 | 0.040 |
| SS | Total potential ammonia oxidation | AOA + AOB + Comammox | / | Sum of guild-associated rates | 0.665 | 0.030 |
| NS | Total potential ammonia oxidation | AOA + AOB + Comammox | / | Sum of guild-associated rates | 0.939 | 0.020 |

**Table S4** Abundances of different nitrifiers and total bacteria in SS and NS sediments. Values are presented as mean ± standard deviation (n = 3).

| Genes | SS | NS |
| --- | --- | --- |
|  | (copies g^-1^ wet sediment) | |
| Comammox *amoA* | (7.17±0.78) ×10^6^ | (2.81±0.86) ×10^6^ |
| AOA *amoA* | (2.15±0.13) ×10^7^ | (1.75±0.18) ×10^7^ |
| AOB *amoA* | (2.45±0.26) ×10^6^ | (1.27±0.12) ×10^6^ |
| *Nitrospira nxrB* | (7.53±0.72) ×10^6^ | (3.79±0.36) ×10^6^ |
| 16S rRNA | (3.94±0.34) ×10^9^ | (2.35±0.07) ×10^9^ |
| Comammox/ammonia-oxidizers | 23.02±3.23% | 13.01±4.41% |
| Nitrifiers/16S | 0.98±0.18% | 1.08±0.17% |
| Comammox/16S | 0.18±0.04% | 0.12±0.04% |

**Table S5.** Treatment-specific regression slopes and derived N₂O and CO₂ rates which are expressed as mg N kg⁻¹ d⁻¹ and mg C kg⁻¹ d⁻¹, respectively.

| Sediment | Process | Slope used as minuend | Slope used as subtrahend | Difference equation | Derived rate | SE |
| --- | --- | --- | --- | --- | --- | --- |
| SS | AOA N₂O rate | TRT III: 0.045 ± 0.005 | TRT IV: 0.030 ± 0.003 | TRT III − TRT IV | 0.016 | 0.008 |
| NS | AOA N₂O rate | TRT III: 1.421 ± 0.061 | TRT IV: 0.253 ± 0.067 | TRT III − TRT IV | 1.169 | 0.107 |
| SS | AOB N₂O rate | TRT II: 0.091 ± 0.001 | TRT III: 0.045 ± 0.005 | TRT II − TRT III | 0.046 | 0.006 |
| NS | AOB N₂O rate | TRT II: 1.976 ± 0.069 | TRT III: 1.421 ± 0.061 | TRT II − TRT III | 0.554 | 0.037 |
| SS | Comammox N₂O rate | TRT I: 0.082 ± 0.004 | TRT II: 0.091 ± 0.001 | TRT I − TRT II | −0.009 | 0.003 |
| NS | Comammox N₂O rate | TRT I: 2.379 ± 0.099 | TRT II: 1.976 ± 0.069 | TRT I − TRT II | 0.403 | 0.061 |
| SS | Total nitrifier N₂O rate | AOA + AOB + Comammox | / | Sum of rates | 0.052 | 0.001 |
| NS | Total nitrifier N₂O rate | AOA + AOB + Comammox | / | Sum of rates | 2.126 | 0.135 |
| SS | AOA CO₂ rate | TRT III: 0.454 ± 0.041 | TRT IV: 0.070 ± 0.007 | TRT III − TRT IV | 0.384 | 0.035 |
| NS | AOA CO₂ rate | TRT III: 0.141 ± 0.009 | TRT IV: 0.089 ± 0.010 | TRT III − TRT IV | 0.052 | 0.019 |
| SS | AOB CO₂ rate | TRT II: 0.393 ± 0.004 | TRT III: 0.454 ± 0.041 | TRT II − TRT III | −0.061 | 0.041 |
| NS | AOB CO₂ rate | TRT II: 0.271 ± 0.015 | TRT III: 0.141 ± 0.009 | TRT II − TRT III | 0.129 | 0.023 |
| SS | Comammox CO₂ rate | TRT I: 0.305 ± 0.034 | TRT II: 0.393 ± 0.004 | TRT I − TRT II | −0.088 | 0.035 |
| NS | Comammox CO₂ rate | TRT I: 0.186 ± 0.013 | TRT II: 0.271 ± 0.015 | TRT I − TRT II | −0.084 | 0.018 |
| SS | Total nitrifier CO₂ rate | AOA + AOB + Comammox | / | Sum of rates | 0.235 | 0.029 |
| NS | Total nitrifier CO₂ rate | AOA + AOB + Comammox | / | Sum of rates | 0.097 | 0.012 |

**Table S6** Quality metrics and summary statistics for the recovered metagenome-assembled genomes (MAGs) of interest.

| MAG |  | bin.86 | bin.140 |
| --- | --- | --- | --- |
| Completeness (%) | | 90.59 | 58.09 |
| Contamination (%) | | 3.76 | 1.94 |
| GC (%) |  | 48.9 | 34.6 |
| Genus (GTDB-tk) | | SZUA-226 | JACEMX01 |
| Family (GTDB-tk) | | Nitrospinaceae | Nitrosopumilaceae |
| N50 |  | 39305 | 8272 |
| Size (bp) |  | 3,067,221 | 952,121 |
| Relative abundance (%) | SS | 1.87 | 4.38 |
|  | NS | 1.98 | 5.87 |
| Expressed genes | | 3,030 (71.8 %) | 1,253 (50.3 %) |

**Table S7.** Physicochemical properties of sediments. Values are presented as mean ± standard deviation (n = 3).

| Parameter | SS | NS |
| --- | --- | --- |
| TON (µg/L) | 8.742 ± 5.151 | 7.014 ± 3.969 |
| Silicate (µg/L) | 191.833 ± 56.717 | 178.324 ± 45.793 |
| TOC (mg/L) | 22.479 ± 1.871 | 21.547 ± 2.946 |
| Ammonia (mg/g) ^***^ | 1.759 ± 0.020 | 1.629 ± 0.007 |
| Nitrate (mg/g) ^***^ | 0.141 ± 0.021 | 0.207 ± 0.007 |
| Nitrite (mg/g) ^***^ | 0.061 ± 0.014 | 0.007 ± 0.002 |
| Phosphate (µg/L) ^*^ | 5.594 ± 1.472 | 4.566 ± 0.959 |
| Salinity (‰) ^***^ | 18.420 ± 0.012 | 18.367 ± 0.037 |
| pH ^**^ | 7.575 ± 0.033 | 7.621 ± 0.034 |

Asterisks denote statistically significant differences between SS and NS based on two-sided paired t-tests (**p* < 0.05, ***p* < 0.01, ****p* < 0.001). TON: total organic nitrogen, TOC: total organic carbon.

**Table S8** Definitions of abbreviations used in Figs. 3, 4, and 6.

| Abbreviation | Definition | Abbreviation | Definition |
| --- | --- | --- | --- |
| Amo | Ammonia monooxygenase | Fru | Fructose PTS system |
| Hao | Hydroxylamine oxidoreductase | APS | Adenosine 5'-phosphosulfate |
| Nxr | Nitrite oxidoreductase | PAPS | 3'-Phosphoadenosine 5'-phosphosulfate |
| Nir | Nitrite reductase (NO-forming) | HK | Hexokinase |
| Nor | Nitric oxide reductase | Pgi | Glucose-6-phosphate isomerase |
| Nar | Nitrate reductase | PFK | Phosphofructokinase |
| Nos | Nitrous oxide reductase | ALD | Fructose-bisphosphate aldolase |
| Cyt554 | Cytochrome c554 | GAPDH/gap | Glyceraldehyde-3-phosphate dehydrogenase |
| mCyt552 | Cytochrome cm552 | TPI | Triosephosphate isomerase |
| Cyt551 | Cytochrome c551 | PGK | Phosphoglycerate kinase |
| Cyt552 | Cytochrome c552 | ENO | Enolase |
| Cyt553 | Cytochrome c553 | PK | Pyruvate kinase |
| CytL | Cytochrome P460 | E | Unknown enzyme |
| NADH | Reduced nicotinamide adenine dinucleotide | Acn | Aconitate hydratase |
| ATP | Adenosine triphosphate | Icd | Isocitrate dehydrogenase |
| ADP | Adenosine diphosphate | Kor | 2-Oxoglutarate ferredoxin oxidoreductase |
| Q | Ubiquinone | Suc | Succinyl-CoA synthetase |
| I | NADH dehydrogenase | Sdh | Succinate dehydrogenase |
| III | Cytochrome bc1 complex | Fum | Fumarate hydratase |
| IV | Cytochrome c oxidase | Mdh | Malate dehydrogenase |
| V | ATP synthase | Acl | ATP-citrate lyase |
| Nrt | Nitrate/nitrite transporter | rbcL | Ribulose-bisphosphate carboxylase large chain |
| Amt | Ammonium transporter | Prk | Phosphoribulokinase |
| Yph | Sugar transport system | Tkt | Transketolase |
| malK | Maltose/maltodextrin ABC transporter | Rpi | Ribose-5-phosphate isomerase |
| PTS | Phosphotransferase system | Gts | Glucose/mannose transport system |
| ScrK | Fructokinase | Man | Phosphomannomutase |
| Gls | Glutaminase | Gmd | GDP-mannose 4,6-dehydratase |
| Sac | Beta-fructofuranosidase/ sucrose degradation | Fcl | GDP-L-fucose synthase |
| Nuo | NADH-quinone oxidoreductase | Pmi | Mannose-6-phosphate isomerase |
| Pet | Ubiquinol-cytochrome c reductase | Mod | Molybdate transport system |
| PetJ | Cytochrome c554 | Cco | Cytochrome c oxidase |
| Qcr | Ubiquinol-cytochrome c reductase | Ndh | NADH dehydrogenase |
| Cox | Cytochrome c oxidase | Msm | Raffinose/stachyose transport system |
| Atp | ATPase | Smo | Sorbitol/mannitol/cellobiose/chitobiose transport system |

**References**

1.	Xu G, Xu X, Yang F, Liu S. Selective inhibition of nitrite oxidation by chlorate dosing in aerobic granules. *J Hazard Mater* 2011;**185**:249–54. https://doi.org/10.1016/j.jhazmat.2010.09.025

2.	Belser LW, Mays EL. Specific inhibition of nitrite oxidation by chlorate and its use in assessing nitrification in soils and sediments. *Appl Environ Microbiol* 1980;**39**:505–10. https://doi.org/10.1128/aem.39.3.505-510.1980

3.	Hink L, Nicol GW, Prosser JI. Archaea produce lower yields of N_2_O than bacteria during aerobic ammonia oxidation in soil. *Environ Microbiol* 2017;**19**:4829–37. https://doi.org/10.1111/1462-2920.13282

4.	Taylor AE, Vajrala N, Giguere AT, Gitelman AI, Arp DJ, Myrold DD et al. Use of aliphatic n-alkynes to discriminate soil nitrification activities of ammonia-oxidizing Thaumarchaea and bacteria. *Appl Environ Microbiol* 2013;**79**:6544–51. https://doi.org/10.1128/AEM.01928-13

5.	Kits KD, Jung M-Y, Vierheilig J, Pjevac P, Sedlacek CJ, Liu S et al. Low yield and abiotic origin of N_2_O formed by the complete nitrifier *Nitrospira inopinata*. *Nat Commun* 2019;**10**:1836. https://doi.org/10.1038/s41467-019-09790-x

6.	Jung M-Y, Well R, Min D, Giesemann A, Park S-J, Kim J-G et al. Isotopic signatures of N_2_O produced by ammonia-oxidizing archaea from soils. *ISME J* 2014;**8**:1115–25. https://doi.org/10.1038/ismej.2013.205

7.	Shen T, Stieglmeier M, Dai J, Urich T, Schleper C. Responses of the terrestrial ammonia-oxidizing archaeon Ca. *Nitrososphaera* viennensis and the ammonia-oxidizing bacterium *Nitrosospira multiformis* to nitrification inhibitors. *FEMS Microbiol Lett* 2013;**344**:121–9. https://doi.org/10.1111/1574-6968.12164

8.	Hynes RK, Knowles R. Inhibition of chemoautotrophic nitrification by sodium chlorate and sodium chlorite: a reexamination. *Appl Environ Microbiol* 1983;**45**:1178–82. https://doi.org/10.1128/aem.45.4.1178-1182.1983

9.	van Ginkel CG, Plugge CM, Stroo CA. Reduction of chlorate with various energy substrates and inocula under anaerobic conditions. *Chemosphere* 1995;**31**:4057–66. https://doi.org/10.1016/0045-6535(95)80007-8

10.	Francis CA, Roberts KJ, Beman JM, Santoro AE, Oakley BB. Ubiquity and diversity of ammonia-oxidizing archaea in water columns and sediments of the ocean. *Proc Natl Acad Sci USA* 2005;**102**:14683–8. https://doi.org/10.1073/pnas.0506625102

11.	Rotthauwe JH, Witzel KP, Liesack W. The ammonia monooxygenase structural gene *amoA* as a functional marker: molecular fine-scale analysis of natural ammonia-oxidizing populations. *Appl Environ Microbiol* 1997;**63**:4704–12. https://doi.org/10.1128/aem.63.12.4704-4712.1997

12.	Zhao Z, Huang G, He S, Zhou N, Wang M, Dang C et al. Abundance and community composition of comammox bacteria in different ecosystems by a universal primer set. *Sci Total Environ* 2019;**691**:146–55. https://doi.org/10.1016/j.scitotenv.2019.07.131

13.	Pester M, Maixner F, Berry D, Rattei T, Koch H, Lücker S et al. *NxrB* encoding the beta subunit of nitrite oxidoreductase as functional and phylogenetic marker for nitrite-oxidizing *Nitrospira*. *Environ Microbiol* 2014;**16**:3055–71. https://doi.org/10.1111/1462-2920.12300

14.	Amann RI, Binder BJ, Olson RJ, Chisholm SW, Devereux R, Stahl DA. Combination of 16S rRNA-targeted oligonucleotide probes with flow cytometry for analyzing mixed microbial populations. *Appl Environ Microbiol* 1990;**56**:1919–25. https://doi.org/10.1128/aem.56.6.1919-1925.1990

15.	Wang Z, Feng K, Wei Z, Wu Y, Isobe K, Senoo K et al. Evaluation and redesign of the primers for detecting nitrogen cycling genes in environments. *Methods Ecol Evol* 2022;**13**:1976–89. https://doi.org/10.1111/2041-210X.13946

16.	Fowler SJ, Palomo A, Dechesne A, Mines PD, Smets BF. Comammox *Nitrospira* are abundant ammonia oxidizers in diverse groundwater-fed rapid sand filter communities. *Environ Microbiol* 2018;**20**:1002–15. https://doi.org/10.1111/1462-2920.14033
